# Disease and Benevolence

**Published:** 1857-06

**Authors:** 


### HALL'S JOURNAL OF HEALTH.

OUR LEGITIMATE SCOPE IS ALMOST BOUNDLESS: FOR WHATEVER BEGETS PLEASURABLE AND HARMLESS FEELINGS, PROMOTES HEALTH; AND WHATEVER INDUCES DISAGREEABLE SENSATIONS, ENGENDERS DISEASE.

VOL. IV.] JUNE, 1857. [NO. VI.

We aim to show how disease may be avoided, and that it is best, when sickness comes, to take no medicine without consulting an educated physician.

### DISEASE AND BENEVOLENCE.

POVERTY, disease, and crime herd together, while thrift, health, and position, are found associated in the same individual. The education of the street leads to the former, the education of the church leads to the latter; by which we mean, that as a matter of civil polity, the most certain exterminator of disease, and crime, and shiftless poverty, is securing to children the benefits of a religious training, of Sunday church-going, of ministerial visitation, and of daily parental counsel on church topics.

Of the two, a thorough collegiate education, with advantages of an European tour, and the being taught to read the Bible by a pious mother and there stop, we have no hesitation in our own mind, in choosing the latter for every child of ours, if we had the option of but one of the two, and would feel an infinitely greater confidence that those children, whether boys or girls, would grow up to be independent, useful, and solid members of society.

It is a common and strong mode of making an assertion, especially in the mouths of wicked men and superficial observers, "How is it that ministers' children are worse than other people's," or, "I have always heard it said that ministers' children are the worst in the world." The sentiment seems to be a conceded fact. But it is utterly destitute of foundation. The son of a minister becoming a derelict, will attract more attention than a thousand equal criminals from the masses; hence the saying.

So far from its being true that the children of ministers, or to have a broader foundation, the children of church members, are worse than other children, it is the very reverse, and, as investigation will show, remarkably so.

A great many pointless sarcasms are thrown out in a certain class of newspapers against "Sunday religion," "Sunday Christians," "church goers," and "being devout on the Sabbath day." It certainly is better to be devout, to be externally religious, one day in a week, than not to be devout at all; better to manifest an external regard for the worship of the Almighty on a Sunday, than to throw off all obligation, and never exhibit anything of the kind.

But, after all, who are the charitable men and women of the times? With here and there a rare exception, they are church-going people, whose habitual practice is to worship in the house of God on the Sabbath day.

We look in vain for the erection of a hospital, the building of a college, the foundation of a professorship, and the institution of beneficiary operations from successful stage actors, play writers, gamblers, horse-racers, and circus riders. But turn to the church-going, Sabbath-abiding merchant, manufacturer, mechanic, farmer, civilian, and you will see in them the friends of good order, the patrons of the deserving, the founders of schools, the builders of churches, and the leading and patron spirits of almost every charitable institution in the land. And that we may not be lacking in facts to sustain our position, we may not do better than to give a list of benefactions made up from a very cursory glancing at our exchanges within a short time past.

1. A gentleman in Detroit proposes to endow The Fine Arts Department of the University of Michigan, and commissions Professor Bradish to proceed to Europe to fulfil this purpose.

2. Henry A. Farrell, of Philadelphia, bequeathed twenty thousand dollars to a Theological Seminary of that city.

3. Miss Elizabeth Galston, of New York, left thirty-five thousand dollars to various benevolent societies.

4. Miss Rachel Morrison, of Louisville, Ky., left two thousand dollars to two institutions of learning.

5. Miss Amelia M. Cone left four thousand five hundred dollars for charitable purposes.

6. The brothers Stuart, members of Dr. Alexander's church in this city, purchased a church which would seat eight hundred and sixty persons, and gave it for the free use of a German Presbyterian congregation, who were too poor to raise the ten thousand dollars required.

7. E. H. Porter, of Memphis, Tennessee, has donated ten thousand acres of land, valued at fifty thousand dollars, to the Presbyterian college at Danville, Kentucky, having previously given ten thousand acres of land to a Methodist college in Tennessee.

8. George W. Johnson, of Louisiana, has directed that two hundred slaves shall be sent to Africa at his own expense, with fifty dollars in money each—equal to an estate of near a quarter of a million of dollars.

9. William Brown gives sixty thousand dollars for a town library, and in addition, appropriates a hundred and fifty thousand dollars towards erecting a free library building.

10. George Peabody gives three hundred thousand dollars in cash, and promises two hundred thousand more, towards establishing an institute in Baltimore for the moral and intellectual culture of its inhabitants.

11. Moses Shepherd left over half a million of dollars to found an insane asylum in Baltimore.

12. Josiah Bradlee sent a check for four thousand dollars, on last New Year's day, for the relief of poor old women, having previously given six thousand dollars for that purpose.

13. John Knickerbocker sent twenty-five hundred dollars, on Christmas day, for benevolent purposes.

14. David Hunt gave fifty thousand dollars to Oakland College lately, having previously given sixty thousand.

15. A New Yorker sent a New Year's present of twenty-five hundred dollars to the American Colonization Society, which will also have a credit, in a few days, for twenty-five thousand more, from Howland, Graham, and others.

16. Mrs. Abbott Lawrence gives a thousand dollars to establish a church.

17. Charles Cook has given twenty-five thousand dollars to a people's college.

18. A lady began nineteen years ago, to give a small sum of money annually to the Bible Society; the more she gives, the more does she seem able to give, until her annual donation is three thousand dollars.

19. A gentleman began, five years ago, to give a hundred dollars a year to the Bible Society, and has gradually increased it until this year it will amount to between sixty-five and seventy-five thousand dollars.

20. Not long ago, a gentleman offered to be one of four who would give twenty-five thousand dollars each, for the establishment of Furnham University.

21. A New York family has given twenty thousand dollars for a clerical school.

22. Mrs. Banyer and Miss Jay (sisters) gave over thirty-three thousand dollars for various charitable objects.

23. Douglass Putnam, of Homer, Ohio, gives twenty thousand dollars to endow Marietta College.

24. W. C. Pierpont bestows thirty-one thousand dollars for the distribution of good books among the people.

25. A gentleman at Nyack, N. Y., has left ninety thousand dollars for benevolent objects, five thousand of which to a man whose whole "life is spent in the truly noble efforts to rescue from crime and elevate the characters of the children of misfortune."

26. Mr. Gray has given to the Boston Athenæum engravings worth twenty-five thousand dollars.

27. Dr. Wales has given his library, worth three thousand dollars, and forty thousand dollars besides, to endow a professorship in a college at Boston.

28. Mrs. Dudley, of Albany, has contributed a hundred thousand dollars for purposes of learning.

Indeed, space would fail to enumerate the thousands and tens of thousands which are steadily flowing into the treasuries of charitable institutions from living church-going and religion-respecting men, such as William H. Dewitt, Alansen Sumner, Robert B. Minturn, James Boorman, Henry Chauncey, John T. Agnew, Mortimer Livingston, the Grinnells, and the Stuart Brothers; while the gifts of Peter Cooper and William B. Astor amount in their life-time to hundreds of thousands of dollars each, for the diffusion of knowledge among men, and for the promotion of the health, comfort, and happiness of their fellow-citizens.

Among the names we have given, we in vain look for one of whom it can be said, He has no respect for the Sabbath; he never enters a church; he makes a jest of religion, and sneers at its ministers. On the contrary, it will be found, with a rare exception, if any, that they and their families are regular attendants on religious worship on the Sabbath day—persons who respect their ministers, and who in all their charities act from principle.

As to the other statement—that the children of clergymen, deacons, and prominent religious professors, are worse than others, it is only necessary, to show its groundlessness, to examine almost any seminary record. Of all the members of the Allegheny Theological Seminary, two years ago, there was but one individual whose father or mother was not a church member.

Of one hundred students at Andover Theological Seminary, only two could say that both their parents were without church membership.

On a recent occasion, when seven young men entered the ministry, it was found that they all had religious parents.

Of 241 families of deacons and ministers, there were 1164 children over fifteen years of age. Of these, 814 appeared to be pious persons, and only fourteen were dissipated; and of these, seven became so after they left their parents.

But what has all this to do with a Journal of Health? Much every way, but chiefly this: the men who honor religion and its ministers, and who are habitual church-goers, are the men mainly to be relied upon in instituting and sustaining those organizations whose objects are human amelioration and human elevation above want, and *disease*, and crime; while the patronizers of the turf, the admirers of the drama, and the frequenters of the card-table, do, by practice and example, cultivate habits of drunkenness, debauchery, and gluttony, and not only ruin themselves, but perpetuate in their offspring bodily diseases and mental temperaments, which, if allowed to mature, would soon corrupt the race and depopulate the world. Therefore, the great practical lesson is, if you want your children to grow up in comparative freedom from idleness, vice, and *disease*, let their evenings be spent at home, and not at the theatre; let their Sundays be spent in religious worship, and not upon the street, or in Sunday "drives" or "excursions." Let them be taught to prefer the school-room to the circus or the race-course, and let their leisure hours be engaged in some instructive employment, some practical handicraft, or some refining amusement, and not at the engine-house, the street corner, the negro minstrelsy, or the ball-room.

We are anxious to be understood, and as anxious not to wound the feelings of any one. We do not mean to say that all church-goers are good men and benevolent. We mean that the natural tendency of an habitual attendance on the house of God is to elevate the character, and that it matters not how bad a man may be, and though he may be far from being a Christian, such regular hearing of a preached gospel will have a greater or less restraint over his character—more or less of a moulding influence for good.

On the other hand, while we admit that there are honorable, and high-minded, and liberal men, who, as husbands, parents, neighbors, and citizens, are worthy of high praise, and yet seldom, if ever, go to church, and look with contempt on religion and its ministers—we know of some such men personally, with whom we would trust our life and everything else with implicit confidence—and there are gamblers, and horse-racers, and stage actors, who have characteristics which we cannot but admire; at the same time, the actual tendencies, the steady results of these things are idleness, thriftlessness, profanity, drunkenness, debauchery, and almost every human degradation.

When William B. Astor gives ten thousand dollars towards enlarging the City Hospital, that there may be more room for those who are brought in with ghastly wounds, and fractured limbs, and broken heads, and follows it up with a hundred thousand dollars for an extension to a public library, where all can come, free of charge, and consult the rarest and costliest books in the world; when Peter Cooper expends three hundred thousand dollars of money, saved from the glue-pot and the iron shop, in erecting a building which fire can never harm, and which, to all appearance, will stand a thousand years, to be occupied as a lecture-room and library in part, where poor young men can come, day or night, without cost, and read and study by the hour, in well-warmed and brightly-lighted apartments, and thus be won from vicious places of amusement and idleness—and in other part, where poor women can come, and, sitting by a comfortable fire and cheery gas-light, sew from early morning to bed-time, without expense to them, and thus be saved from the disease-engendering foul rooms, basement dwellings, and cold, damp houses, which make the early graves of so many of the industrious poor; when Robert Lenox devotes the annual income of his inherited millions, excepting what is necessary for his own personal expenses, to every well-directed benevolence which meets his sympathies (and long may he, and the Stuarts, and other *living* benefactors, live to continue their good doings!)—when men like these bestow their charities, there is no return to them but the sweet, abiding consciousness of good done. That good inures to others—to persons whom they have never seen, to save them from suffering, and to elevate them in the social scale. But the horse-racer, with his boast of improving the breed of that noble animal, how long, think you, would he continue in that occupation if he were prohibited from betting on the race, and thus all chance of pecuniary profit be cut off? And how long would the stage player walk the boards to "elevate the drama," were he to be limited to the salary of the self-denying Methodist circuit rider, and more especially if the drinking bouts and other immoralities of the green-room were abrogated? And as to the remorseless gambler, he makes no claim to human or even animal good, outside of himself, as an apology for his occupation. He calls it a "gentlemanly amusement," the trickeries of the trade included—the studied deceptions essential to success. As to honor among gamblers, what is it? No rascality is dishonorable to them, except that which is so vulgar as to be found out.

As to the influences which they all three exert together—the theatre, the race-course, and the gaming table—the very atmosphere of their locality is polluted. More—it is pestilential. All, both old and young, are corrupted by it; attracting to them all that is mean, and low, and vile, and driving from them in a panic whatever was quiet, orderly, and refined that happened to be included in their precincts; so that drunkenness, rioting, and obscenity, are left without a check, and all grow, and fester, and rankle, and rot together.

That we may not seem to be alone in these views, we close with a paragraph from *Harper's Weekly*, which is not a religious but a secular paper: "The drama in America, from its inaptitude and rudeness, does not attract the taste of the refined, and from its grossness positively repels the sentiment of the moral. Its influence is confined to corrupting the inexperienced, or fixing the habits of the vicious. Entirely a foreign thing in its nature and management, the so-called American drama has never touched the national sympathy, and accordingly its bad effects are, fortunately, very restricted. It is only, after all, a kind of chiffonier or rag-picker, which fumbles in the filth of society, and gathers up the loose ends and dirty shreds of humanity. These, however, are worth the trial of purification, and it becomes social reformers to attempt it. What better proof can you have of the evil influences of the theatre than the rapid corruption which ensues in a neighborhood on the raising of one of these temples of vice? No sooner is the flaming poster stuck up, the doors opened, and the gas lighted, than decency flies it, as health would a plague-spot. The erection of a new theatre in a previously respectable quarter of one of our cities, is well known to destroy that quarter for any future decency of life. The private house is turned into the bagnio; the shop of honest trade into the faro, saloon, or bar room, and the playhouse stands a spectacle of vice, supported by its congenial aids of rowdyism, gambling, drunkenness, and prostitution. Verily, the national taste and morality do well in scorning the 'Theatre and its Friends.'"

